# Constipation Symptoms in Multiple System Atrophy Using Rome Criteria and Their Impact on Personalized Medicine

**DOI:** 10.3390/jpm12050838

**Published:** 2022-05-20

**Authors:** Takayasu Mishima, Shinsuke Fujioka, Miki Kawazoe, Kotoe Inoue, Hisatomi Arima, Yoshio Tsuboi

**Affiliations:** 1 Department of Neurology, School of Medicine, Fukuoka University, Fukuoka 814-0180, Japan; mishima1006@fukuoka-u.ac.jp (T.M.); shinsuke@cis.fukuoka-u.ac.jp (S.F.); kotoei@fukuoka-u.ac.jp (K.I.); 2 Department of Preventive Medicine and Public Health, School of Medicine, Fukuoka University, Fukuoka 814-0180, Japan; miki1024@fukuoka-u.ac.jp (M.K.); harima@fukuoka-u.ac.jp (H.A.)

**Keywords:** multiple system atrophy, Parkinson’s disease, constipation, irritable bowel syndrome, Rome III, Rome IV, Rome criteria

## Abstract

Constipation is one of the most common non-motor symptoms in multiple system atrophy (MSA); however, it has not been evaluated according to the standard diagnostic criteria for constipation in patients with MSA. We evaluated the characteristics of constipation in patients with MSA by using Rome criteria (Rome III), which has been validated and the widely used for gastrointestinal disorders. Fifty-one patients with MSA (29 female) were enrolled in the study. Based on the Rome III criteria, constipation was diagnosed in 29 patients (56.9%); irritable bowel syndrome was not detected. Thirty-seven patients (72.5%) were aware of their constipation. The most common constipation symptom was the sensation of anorectal obstruction (68.6%). Patients’ self-awareness of constipation was most strongly correlated to the sensation of incomplete evacuation (odds ratio: 7.377, 95% confidence interval: 1.402–38.817). The number of constipation-related symptoms was correlated with the total levodopa equivalent dose (*p* < 0.05). Rome criteria, which can detect various constipation symptoms, are useful for evaluating constipation in MSA, and these findings may greatly impact personalized medicine.

## 1. Introduction

Constipation is one of the most common non-motor symptoms seen in patients with extrapyramidal disorders [1]. The incidence of constipation in Parkinson’s disease (PD) has been reported in several studies, showing that constipation is prevalent in PD patients and has a substantial impact on quality of life [2,3]. Straining during defecation is the most common feature in patients with PD, and we have previously shown that this symptom is most closely related to their self-awareness of constipation based on Rome criteria (Rome III) [4]. Rome criteria are the most used standard criteria for diagnosing functional gastrointestinal disorders; Rome III diagnostic criteria were published in 2006 [5,6]. Rome criteria can diagnose functional bowel disorders, including irritable bowel syndrome (IBS) and functional constipation, and can detect detailed symptoms of constipation, such as straining during defecations; lumpy or hard stools of defecations; sensation of incomplete evacuation; sensation of anorectal obstruction/blockage; manual maneuvers to facilitate defecations; and fewer than three defecations per week.

Multiple system atrophy (MSA) is a sporadic and progressive neurodegenerative disease categorized as an α-synucleinopathy [7]. Other α-synucleinopathies include conditions such as PD, dementia with Lewy bodies, and pure autonomic failure. Clinically, MSA is characterized by a combination of autonomic dysfunction, parkinsonism, cerebellar ataxia, and pyramidal signs. MSA is classified into two categories: MSA-P, predominantly parkinsonism; and MSA-C, predominantly cerebellar ataxia [8]. MSA may be indistinguishable from PD in the early stages of the disease because MSA causes parkinsonism, autonomic dysfunction, rapid eye movement (REM) sleep disorder, depression, and cognitive impairment, as well as PD [9,10]. Similar to the observations in PD, constipation is a common autonomic disorder in MSA [11]. However, it has been reported that PD and MSA have different preganglionic and postganglionic fiber dysfunction in the autonomic nervous system and that dysuria in both diseases also has different symptoms even at an early stage [12,13]. Therefore, the differences in the symptoms of constipation may also help in the early diagnosis of these seemingly similar neurodegenerative disorders. However, despite this potential, the reports on the characteristics of constipation in MSA are scarce. To address this knowledge gap, we aimed to assess the characteristics of constipation in patients with MSA using the Rome criteria (Rome III).

## 2. Methods

### 2.1. Participants and Ethical Standards

The present study is a cross-sectional analysis performed at the Fukuoka University Hospital. A total of 51 patients who visited our department of neurology between April 2017 and November 2021 were consecutively enrolled in this study. All participants were of Japanese origin and clinically diagnosed by certified movement disorder specialists (TM, SF, and YT) as having probable MSA based on the consensus criteria of MSA [8]. Patients with other parkinsonian disorders were excluded. Demographics and patient clinical data, including the age, disease duration at the time of examination in this study, gender, neurological findings, and the usage of dopaminergic medications, were prospectively evaluated. The Japanese version of the Rome III questionnaire was administered in an in-person manner [4,14]. Cognitive assessments were completed utilizing the Mini-Mental State Examination (MMSE). The daily dosage of anti-parkinsonian medications was converted to a total levodopa equivalent dose (LED) according to previously published protocols [15]. The use of anticholinergics and laxatives was evaluated due to their potential for changing bowel habits. This study was approved by the Institutional Review Board of the Fukuoka University (No. 15-5-06), and informed consent was obtained from all participants.

### 2.2. Data Analysis

Student’s *t*-test (for quantitative variables) and Fisher’s exact test (for qualitative variables) were used to compare the demographic differences between the patients with constipation and patients without constipation and IBS. Multiple logistic regression analysis was used to estimate the odds ratio (OR) and 95% confidence interval (CI) to assess the association between the self-awareness of constipation and the symptoms of constipation. Spearman’s rank correlation coefficient was used to evaluate the association between the frequency of constipation symptoms and baseline data. All analyses were performed using SPSS (version 26, Armonk, NY, USA) and SAS (version 9.4, Cary, NC, USA), and the statistical significance level was set at two-tailed *p* < 0.05.

## 3. Results

### 3.1. Patient Characteristics

MSA-C and MSA-P were detected in 22 patients (43.1%) and 29 patients (56.9%), respectively. Of the 51 patients, 22 (43.1%) were males. The mean age and mean disease duration at the time of evaluation were 65.6 ± 10.1 and 4.3 ± 2.7 years, respectively. The mean total MMSE score was 26.8 ± 3.0. The average LED was 230.7 ± 309.9 mg/day. The number of patients who used anticholinergics and laxatives was 0 (0%) and 21 (39.2%), respectively. Characteristics of the patients with constipation and those without constipation and IBS are shown in Table 1. Constipation and IBS were detected in 29 patients (56.9%) and 0 patient (0%), respectively. The constipation group had more laxative use than the non-constipation group (*p* < 0.05) (Table 1).

### 3.2. Characteristics of Constipation in MSA Patients

The most common symptom related to constipation was the sensation of anorectal obstruction/blockage (68.6%). Thirty-seven patients (72.5%) reported self-awareness of constipation. Among constipation-related symptoms, patients’ self-awareness of constipation was most strongly associated with the sensation of incomplete evacuation (OR: 7.377, 95% CI: 1.402–38.817) (Table 2). The number of constipation symptoms was correlated with the total LED (*p* < 0.05) (Table 3). Characteristics of the patients with MSA-C and MSA-P are shown in Table 4. Comparison of constipation symptoms among groups of MSA-C and MSA-P are listed in Table 5. The MSA-P group had more constipation than the MSA-C group (*p* < 0.05) (Table 5) and more LED and laxative use than the MSA-C group (*p* < 0.05) (Table 4). Concerning symptoms of constipation, MSA-P had a higher frequency of sensation of incomplete evacuation, sensation of anorectal obstruction, and fewer defecations than MSA-C (*p* < 0.05) (Table 5).

## 4. Discussion

The present study is the first report to evaluate the prevalence and characteristics of constipation in MSA patients based on the Rome III diagnostic criteria. The frequency of constipation in MSA has been reported to be approximately 80% [7,16,17,18], while it was 56.9% in the present study. Our study tended to have a lower frequency of constipation using Rome criteria compared to previous reports. It may be due to a more stringent diagnosis of constipation under Rome criteria [4].

Although the characteristics of constipation in MSA have been reported to be similar to those in PD, the efforts aimed at differential diagnosis have been lacking [19]. A comparison of this MSA cohort with our previously reported study in PD patients [4] clearly suggests that the two conditions differ in the characteristics of functional bowel disorders. First, the frequency of constipation was 27.1% [4] in patients with PD and 56.9% in patients with MSA, indicating that constipation is more frequent in MSA than in PD. Second, the self-awareness of constipation in patients with PD was 81.4% [4], whereas it was 72.5% in patients with MSA. There was a large dissociation between the self-awareness of constipation and the diagnosis of constipation in patients with PD, while the dissociation was smaller in patients with MSA. Previous studies have reported that the frequency of constipation was 90% in patients with PD and 80% in patients with MSA [7,16,17,18], a trend similar to the frequency of self-awareness of constipation in the present study. Third, the diagnosis of constipation as per the Rome criteria requires the presence of at least two constipation symptoms probed by the questionnaire [5,6,14]. Therefore, the greater frequency of constipation in MSA as opposed to PD, when diagnosed by the Rome III criteria, may suggest that constipation in MSA is generally more severe than that in PD. Fourth, the present study revealed that the most common constipation symptom in patients with MSA was the sensation of anorectal obstruction/blockage. The self-awareness of constipation in patients with MSA was most strongly correlated with the sensation of incomplete evacuation. In contrast, straining was the most common and correlated symptom with patients’ self-awareness of constipation in patients with PD [4]. These results indicate that anorectal dysfunction is more common or severe in MSA than in PD [19].

The difference in the characteristics and severity of constipation in the two disorders may be related to the differences in autonomic pathology. In particular, the two common constipation symptoms in patients with MSA—the sensation of anorectal obstruction/blockage and the sensation of incomplete evacuation—may be due to the underlying autonomic dysfunction in the spinal cord, especially since MSA affects the sacral Onuf’s nucleus early in the disease course [19]. The most important difference in functional bowel disorders between MSA and PD is the presence or absence of IBS. No IBS was detected in MSA patients in this study. This fact contrasts with our previous study showing that IBS is common in PD [4]. Large-scale cohort studies and meta-analyses have shown that IBS is a risk factor for PD [20,21]. Therefore, the presence of IBS may help distinguish PD from MSA. The present study indicated that the number of constipation symptoms, which indicates the severity of constipation, was correlated with LED in patients with MSA, consistent with the observations in patients with PD [4]. Although dopaminergic treatment is effective to some degree in some patients with MSA [22], its potency may be limited. Because dopaminergic treatment may increase the severity of constipation in patients with MSA due to inhibiting the movement of the intestinal tract [23], careful consideration of dosage is recommended.

Growing evidence suggests that α-synucleinopathy in the enteric nervous system contributes to the gastrointestinal dysfunction associated with PD [19,24]. In PD, α-synuclein aggregates of the myenteric plexus may cause functional bowel disorders. In contrast, MSA primarily affects the central nervous system, such as the brain and spinal cord, but not the enteric nervous system [19]. Recently, the relationship between the accumulation of α-synuclein and gut dysbiosis has been attracting increased attention [24,25,26,27]. Nishiwaki et al. analyzed the gut microbiota of PD patients worldwide [24]. They reported an increase in *Akkermansia*, intestinal bacteria responsible for the degradation of the mucin layer, and a decrease in *Faecalibacterium* and *Roseburia*, the short-chain fatty acid-producing bacteria. It has been suggested that the increased intestinal wall permeability due to the intestinal wall mucin degradation may promote the accumulation of α-synuclein aggregates in the intestinal plexus, and the decrease in short-chain fatty acids may lead to the progression of PD due to the inability to suppress inflammation in the central nervous system. Although IBS has also been associated with gut microbiota [28,29], the relationship between PD and IBS with respect to gut microbiota needs to be clarified. Although a few reports have examined the α-synuclein accumulation in the enteric nervous system in MSA [19], additional studies examining the role of gut microbiota in MSA pathophysiology are warranted. These studies may elucidate the causative factors in the pathogenesis of MSA and reveal its similarities and differences with PD.

There are some limitations of the current study. First, this study was a short-term, uncontrolled, single-center study with a small sample size. Second, variables that could potentially influence stool consistency and bowel habits, including smoking habits [30], coffee intake [30], water intake [31], insoluble fiber intake [32], probiotics [33], beta-blockers [34], and other constipation-inducing drugs [34] were not investigated in this study. Third, the study did not entail a colonoscopy to exclude a diagnosis of gastrointestinal disorders such as anatomic, metabolic, inflammatory, or neoplastic causes [35]. Functional evaluation, including a colon transit time examination, might also be useful to rule out alternative causes of bowel disorders [36]. Finally, this study utilized Rome III as the diagnostic criteria for constipation and not Rome IV, published in 2016 [37]. The Rome III and Rome IV diagnostic criteria do not include abdominal discomfort in the definition of IBS, thereby changing the symptom frequency threshold. However, there is no significant difference in the diagnosis of constipation [37]. This study also showed that MSA-P had a higher frequency of constipation than MSA-C, consistent with previous studies [38]. A detailed analysis of gastrointestinal symptoms may help differentiate PD from MSA, and in future studies, using Roman IV in patients with larger-scale PD and MSA is desirable.

Despite these limitations, the present study reveals that the Rome criteria can be useful for personalized medicine for constipation in MSA. The use of Rome criteria has applications in determining the therapeutic efficacy of laxatives in MSA. Furthermore, multinational, multicenter, and longitudinal studies are required to explore the pathophysiology of gastrointestinal symptoms in MSA patients.

## Figures and Tables

**Table 1:** Comparison of demographic and clinical characteristics among groups of multiple system atrophy patients with and without constipation.

	Patients without Constipation (*n* = 22)	Patients with Constipation (*n* = 29)	*p* Value
Age (years)	65.3 ± 9.5	65.2 ± 11.1	0.434
Male (%)	40.9	56.5	0.503
Disease duration (years)	4.0 ± 2.7	4.9 ± 3.3	0.328
MMSE	26.6 ± 3.6	26.9 ± 2.5	0.718
LED (mg/day)	189.8 ± 344.3	244.3 ± 284.8	0.497
Laxative use (%)	22.7	55.2	0.019
Anti-cholinergic use (%)	0	0	n.s.

Data are presented as the mean ± SD. LED, levodopa equivalent dose; MMSE, Mini-Mental State Examination; n.s., not significant.

**Table 2:** Frequencies of constipation symptoms based on Rome III diagnostic questionnaire and their association with patient’s awareness of constipation in multiple system atrophy cohort.

Question Items	Frequency (%)	OR	95% CI
Straining	61.9	2.404	0.575–10.040
Lumpy or hard stools	47.0	2.775	0.668–11.532
Sensation of incomplete evacuation	51.0	7.377	1.402–38.817
Sensation of anorectal obstruction	68.6	2.462	0.583–10.398
Manual maneuvers	13.7	Not calculated	Not calculated
Fewer defecations	33.3	3.473	0.615–19.616

Patients identified straining, lumpy or hard stools, sensation of incomplete evacuation, sensation of anorectal obstruction, and manual maneuvers as their constipation symptoms if they occurred in at least 25% of defecations. Fewer defecations refer to fewer than three defecations per week. OR, odds ratio; CI, confidence interval.

**Table 3:** Correlation analysis of the number of constipation symptoms and baseline data.

	r	*p* Value	95% CI
Age (years)	0.035	0.809	−0.252–0.321
Disease duration (years)	0.205	0.149	−0.066–0.477
MMSE	−0.124	0.387	−0.430–0.182
LED (mg/day)	0.370	0.008	0.138–0.602

LED, levodopa equivalent dose; MMSE, Mini-Mental State Examination; CI, confidence interval.

**Table 4:** Comparison of demographic and clinical characteristics among groups of MSA-C and MSA-P.

	MSA-C (*n* = 22)	MSA-P (*n* = 29)	*p* Value
Age (years)	63.4 ± 10.8	67.0 ± 9.6	0.505
Male (%)	36.3	48.3	0.569
Disease duration (years)	4.6 ± 3.3	4.3 ± 2.8	0.466
MMSE	27.3 ± 2.7	26.4 ± 3.2	0.956
LED (mg/day)	40.9 ± 140.3	377.1 ± 326.2	0.032
Laxative use (%)	18.2	62.0	0.002

Data are presented as the mean ± SD. MSA-C, multiple system atrophy, predominant cerebellar ataxia; MSA-P, multiple system atrophy, predominant parkinsonism; LED, levodopa equivalent dose; MMSE, Mini-Mental State Examination.

**Table 5:** Comparison of constipation symptoms among groups of MSA-C and MSA-P.

	MSA-C (*n* = 22)	MSA-P (*n* = 29)	*p* Value
Constipation (%)	40.9	69.0	0.045
Straining (%)	50.0	72.4	0.101
Lumpy or hard stools (%)	36.4	55.2	0.183
Sensation of incomplete evacuation (%)	31.8	65.5	0.017
Sensation of anorectal obstruction (%)	50.0	82.8	0.013
Manual maneuvers (%)	9.1	17.2	0.402
Fewer defecations (%)	18.2	44.8	0.046
